# Supplementary material for: Treatment of Acquired Deforming Hypertonia with Botulinum Toxin in Older Population: A Retrospective Study
Source: Toxins (Basel). 2024 Aug 16;16(8):365. doi: 10.3390/toxins16080365 (PMC11359055; doi:10.3390/toxins16080365)
Supplement: Supplementary file 1 [file toxins-16-00365-s001.zip › toxins-3132810-supplementary.pdf]

toxins

Table S1. The AGGIR scale.

English translation of an extract of the government document published in French available

https://www.pour-les-personnes-agees.gouv.fr/preserver-son-autonomie-s-informer-et-anticiper/perte-d-autonomie-evaluation-et-droits/comment-fonctionne-la-grille-aggir

15 August 2024

The six GIR comprising the AGGIR scale may be described as follows:

GIR 1 corresponds to older persons who are confined to bed, whose mental functions are severely impaired, and for whom the continuous presence of caregivers is essential.

GIR 2 includes two major categories of older persons:

- those who are confined to bed or an armchair, whose mental functions are not fully impaired, but who need assistance for most everyday activities,
- those whose mental functions are impaired, but who have maintained their mobility.

GIR 3 essentially corresponds to older persons who have fully or partially maintained their mental autonomy and partially their locomotor autonomy, but who need assistance for their bodily care several times a day.

GIR 4 comprises two categories of older persons:

- those who cannot get up, lie down, or sit down alone, but once standing can move about within their place of residence; they may sometimes need help for washing and dressing. The great majority of these persons can eat unaided.
- those who have no difficulties with respect to mobility, but need help for bodily care and for meals.

GIR 5 comprises persons capable of moving about alone within their place of residence, who can eat and dress themselves alone, but who may need occasional help for domestic activities (preparing meals, household chores, etc.).

GIR 6 comprises persons who are autonomous with respect to all discriminatory acts of everyday life*, but who may need occasional help for domestic activities.

*The 10 defined discriminatory variables comprise:

- coherence (communication, conversation, sensible behavior)
- orientation (in time and place)
- washing
- dressing
- eating
- urinary and fecal hygiene
- positional transitions (getting up, lying down, sitting down)
- moving around within one's place of residence
- moving around outside one's place of residence (without the use of means of transport)
- communication over a distance (use of a telephone, alarm, etc.).

Table S2. Study questionnaire:.

Telephone-administered questionnaire evaluating the efficacy and potential adverse effects of botulinum toxin injections in elderly patients with ADH

Investigator: Patient no.: Person replying to the questionnaire:

1. **What were the goals targeted by the injections?**<br>- To relieve pain<br>- To facilitate dressing and/or daily care<br>- To facilitate limb mobilization<br>- To prevent or cure skin complications (pressure ulcers or moisture-associated skin damage)<br>- To facilitate comfortable positioning<br>- Other: (please specify)	2. **In your opinion, has the treatment had an impact on these goals?**<br>- Not<br>- Below expectations<br>- Standard performance<br>- Exceeded expectations<br>- Much better than expected
3. **Do you think these injections improved your (the patient's) quality of life?**<br>- Yes, significantly<br>- Yes, moderately<br>- No, not at all	4. **Do you think these injections reduced the time needed by care-givers to accomplish daily care or the difficulty of this task?**<br>- Yes, significantly<br>- Yes, moderately<br>- No, not at all
5. **On a scale of 0 to 10, how would you rate the overall benefit of these injections (0: no benefit; 10: maximum benefit)?**	6. **Did you (did the patient) perform the mobilization exercises alone?**<br>- Yes<br>- No<br>- I don't know
7. **Were the mobilization exercises performed with the help of your (the patient's) care-givers or family members?**<br>- Yes<br>- No<br>- I don't know	8. **If not, why?**<br>- Exercises not understood, not remembered, or impossible alone<br>- I (the patient) refused<br>- Lack of time<br>- Other: (please specify)
9. **Do you think these injections should be repeated?**<br>- Yes<br>- No<br>- I don't know	10. **Did you notice any side effects following the injections?**<br>- No<br>- Hematoma at the site of injection<br>- Itching at the site of injection<br>- Reddening at the site of injection<br>- Pain at the site of injection<br>- Other: (please specify)
11. **Did you (did the patient) experience any side effect related to botulinum toxin after the injection ?**	12. **Have you (has the patient) presented any non-serious side effect since the injection?**

- Localized muscle weakness affecting the limb injected<br>- Difficulty in swallowing<br>- Generalized muscle weakness<br>- Tiredness<br>- Other: (please specify)	
